# Characterization of multi-domain postoperative recovery trajectories after cardiac surgery using a digital platform

**DOI:** 10.1038/s41746-022-00736-0

**Published:** 2022-12-24

**Authors:** Makoto Mori, Sanket S. Dhruva, Arnar Geirsson, Harlan M. Krumholz

**Affiliations:** 1 Division of Cardiac Surgery, Yale School of Medicine, New Haven, CT USA; 2 Center for Outcomes Research and Evaluation, Yale New Haven Hospital, New Haven, CT USA; 3 Department of Medicine, University of California San Francisco School of Medicine, San Francisco, CA USA; 4 Section of Cardiology, San Francisco VA Medical Center, San Francisco, CA USA; 5 Section of Cardiovascular Medicine, Department of Internal Medicine, Yale School of Medicine and the Department of Health Policy and Management, Yale School of Public Health, New Haven, CT USA

**Keywords:** Outcomes research, Cardiovascular diseases

## Abstract

Understanding postoperative recovery is critical for guiding efforts to improve post-acute phase care. How recovery evolves during the first 30 days after cardiac surgery is not well-understood. A digital platform may enable granular quantification of recovery by frequently capturing patient-reported outcome measures (PROM) that can be clinically implemented to support recovery. We conduct a prospective cohort study using a digital platform to measure recovery after cardiac surgery using a PROM sent every 3 days for 30 days after surgery to characterize recovery in multiple domains (e.g., pain, sleep, activities of daily living, anxiety) and to identify factors related to the patient’s perception of overall recovery. We enroll patients who underwent cardiac surgery at a tertiary center between January 2019 and March 2020 and automatically deliver PROMs and reminders electronically. Of the 10 surveys delivered per patient, 8 (IQR 6–10) are completed. Patients who experienced postoperative complications more commonly belong to the worst overall recovery trajectory. Of the 12 domains modeled, only the worst anxiety trajectory is associated with the worse overall recovery trajectory membership, suggesting that even when patients struggle in the recovery of other domains, the patient may still feel progress in their recovery. We demonstrate that using a digital platform, automated PROM data collection, and characterization of multi-domain recovery trajectories is feasible and likely implementable in clinical practice. Overall recovery may be impacted by complications, while slow progress in constituent domains may still allow for the perception of overall recovery progression.

## Introduction

Understanding how patients recover from cardiac surgery is important for preoperative patient counseling^1^, resource allocation^2^, and post-acute strategies^3^. However, few studies have evaluated the recovery after cardiac surgery from the patient perspective, using patient-reported outcome measures (PROMs) administered at a high frequency after hospital discharge^4–6^. Frequent PROM data collection in the early period after discharge can capture the recovery trajectory but can burden the participants and may be resource-intensive. Digital platforms leveraging smartphones and tablets may facilitate frequent data collection, reducing personnel time and effort and increasing patient engagement^7^. Assessing the feasibility of a less resource-intensive way of PROM data collection has important implications in informing a larger-scale implementation of such an approach to propel the field of postoperative recovery research and develop applications in routine clinical practice.

Recovery is a complex phenomenon with an interplay of physiologic, physical, and mental responses to surgery. A comprehensive understanding of postoperative recovery requires measuring PROMs across various domains, including pain, ability to complete activities of daily living (ADL), and mental well-being^8^. While the recovery in each domain likely contributes to the patient’s perception of overall recovery, this interplay and factors associated with the patient’s perception of overall recovery progress are not well-understood.

We conducted a study to evaluate the feasibility of using a digital platform to collect data, aiming to include diverse patients by simplifying enrollment and automating data collection^9^. We demonstrate that using a digital platform, automated PROM data collection, and characterization of multi-domain recovery trajectories is feasible and likely implementable in clinical practice. In the first 30 days after cardiac surgery, overall recovery may be impacted by complications, while slow progress in constituent domains may still allow for the perception of overall recovery progression.

## Results

### Patient characteristics

The median age (IQR) of the 80 patients analyzed was 64 (57, 70) years, of which 20 (25%) were women and 72 (90%) were of the Caucasian race, which are comparable demographics distribution to our center’s consecutive CABG patients^10^. Forty (50%) patients underwent CABG, 18 (22%) underwent AVR, and 27 (33%) underwent mitral valve surgery. There were 7 (9%) redo sternotomy cases, and 24 (30%) were operated on urgent bases (Table 1).

**Table 1:** Patient characteristics by the overall recovery trajectories.

Characteristics	Good recovery (*N* = 68)	Poor recovery (*N* = 12)	*P*
Age	65 (58, 71)	60 (54, 62)	0.08
Woman	18 (26%)	2 (17%)	0.7
Race			0.7
Black	4 (5.9%)	0 (0%)	
White	61 (90%)	11 (92%)	
Other	3 (4.4%)	1 (8.3%)	
Diabetes	24 (35%)	2 (17%)	0.3
Hypertension	50 (74%)	6 (50%)	0.2
Liver disease	6 (8.8%)	0 (0%)	0.6
Creatinine (mg/dL)	0.96 (0.84, 1.19)	0.97 (0.84, 1.08)	0.9
Cerebrovascular disease	9 (13%)	0 (0%)	0.3
Prior myocardial infarction	18 (26%)	5 (42%)	0.3
Heart failure	22 (32%)	3 (25%)	0.7
Ejection fraction (%)	60 (55, 63)	59 (43, 63)	0.5
Status			>0.9
Elective	47 (69%)	9 (75%)	
Urgent	21 (31%)	3 (25%)	
Re-do operation	6 (8.8%)	1 (8.3%)	>0.9
CABG	34 (50%)	6 (50%)	>0.9
Aortic surgery	4 (5.9%)	0 (0%)	>0.9
AVR	15 (22%)	3 (25%)	>0.9
MV surgery	23 (34%)	4 (34%)	>0.9
Robotic approach	15 (22%)	2 (17%)	>0.9
Postoperative length of stay (days)	5 (4, 6)	6.5 (4, 7.75)	0.06
Any complications	24 (35%)	9 (75%)	**0.023**
Surgical site infection	2 (2.9%)	1 (8.3%)	0.4
Reoperation for bleeding	0 (0%)	2 (17%)	**0.021**
Sepsis	0 (0%)	0 (0%)	
Stroke	0 (0%)	0 (0%)	
TIA	0 (0%)	0 (0%)	
Prolonged ventilatory support	0 (0%)	2 (17%)	**0.021**
Pneumonia	0 (0%)	1 (8.3%)	0.15
VTE	0 (0%)	1 (8.3%)	0.15
Renal failure	0 (0%)	0 (0%)	>0.9
Readmission	4 (5.9%)	2 (17%)	0.2
30-day survival	68 (100%)	12 (100%)	>0.9

*P* values in bold indicate *p* < 0.05. For significance testing, chi-squared test and Wilcoxon rank-sum test were used for categorical and continuous variables, respectively. *CABG* coronary artery bypass graft surgery, *AVR* surgical aortic valve replacement, *MV* mitral valve, *TIA* transient ischemic attack, *VTE* venous thromboembolism.

### Response rate and reasons for difficulty responding

Of the 10 surveys delivered, the median number of responses was 8 (IQR: 6,10). There were 47 patients (59%) who responded to 8 or more surveys, 18 (23%) who responded to between 5 and 8 surveys, and 15 (19%) who responded to 4 or fewer surveys. Including the 12 patients who responded to only one survey (excluded from the analysis), we attempted to contact 27 patients who responded to 4 or fewer surveys, 19 of whom we could reach. Of the 19, the most common patient-reported reason for low response rate had other priorities (*n* = 7), challenge responding due to clinical conditions (*n* = 5), the survey being too long or too frequent (*n* = 3), survey software issue (*n* = 2), and having difficulty finding the survey in the inbox (*n* = 2). Patient characteristics did not differ significantly between those who responded <80% versus ≥80% (Supplemental Table 1). The average time spent on survey completion was 5.2 ± 1.5 min.

### Group-based trajectory model

**Fig. 1:**
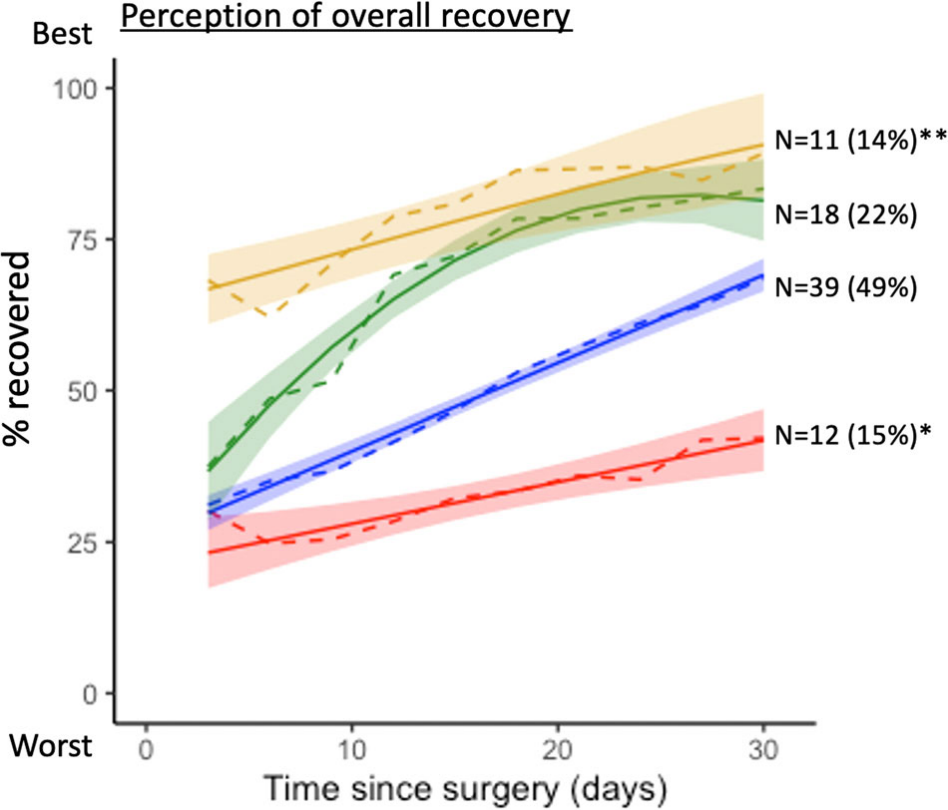
Trajectories of patient perception of overall recovery. The figure shows four trajectories of overall recovery on the patient-reported scale of 0 to 100%. The trajectory groups were identified by fitting the group-based trajectory model. The yellow curve depicts the best recovery trajectory, including 11 patients, and the red curve depicts the worst recovery trajectory, including 12 patients. Trajectory with a single asterisk (*) denotes the worst recovery trajectory and the trajectory with double asterisks (**) denotes the best trajectory.

For the patient perception of overall recovery, the group-based trajectory model with four trajectories yielded the best fit based on the Bayesian information criterion (BIC) value (Fig. 1). There were 11 patients who were classified into the best recovery trajectory (Fig. 1, yellow curve), depicting a high percentage of perceived overall recovery since the first measurement and a persistent increase in the perceived overall recovery throughout the 30-day period. There were 12 patients who were classified into the worst recovery trajectory (Fig. 1, red curve), depicting low perceived overall recovery throughout the first 30 days.

**Fig. 2:**
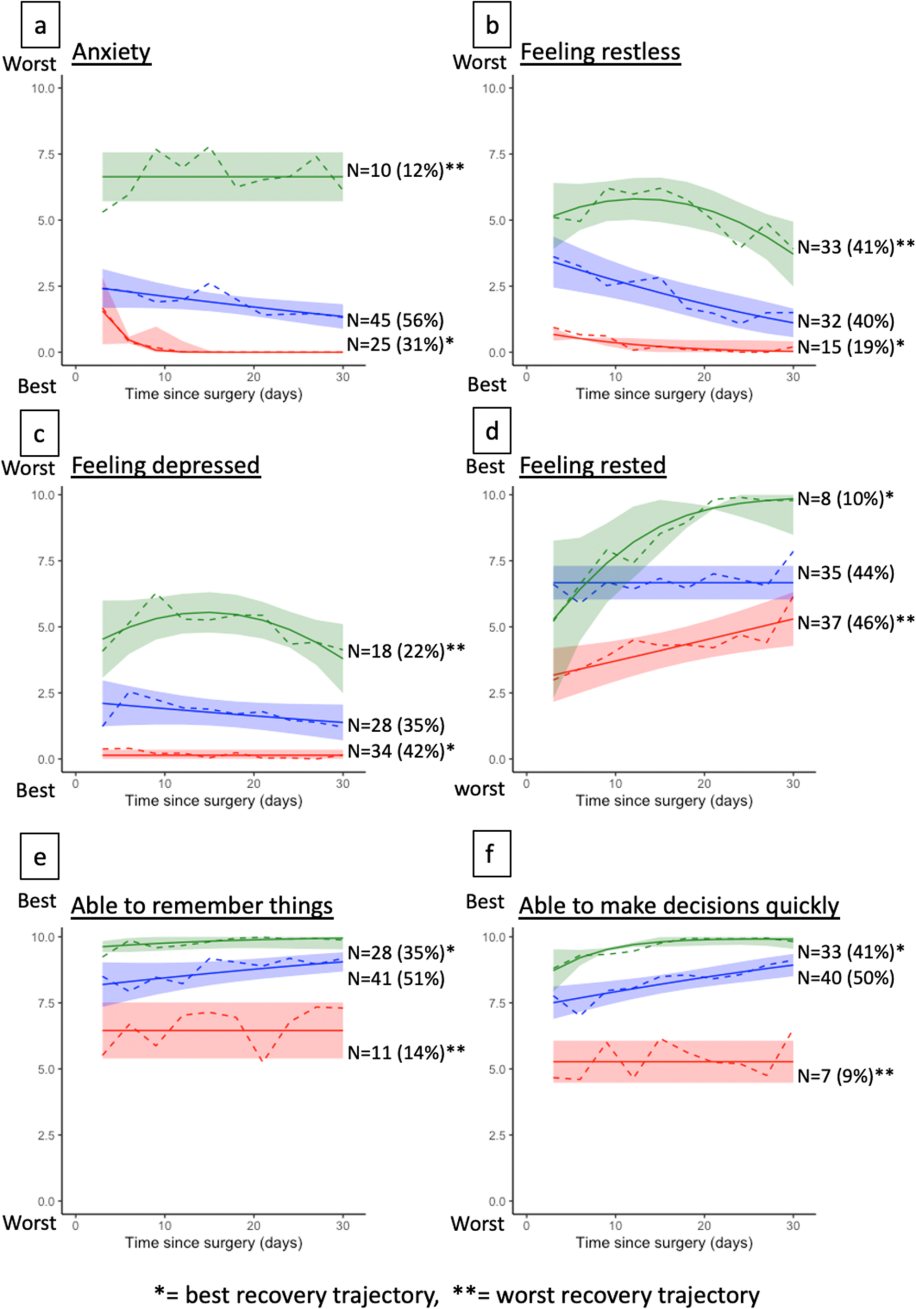
Trajectories of recovery by emotive and cognitive domains. The figure shows trajectories of recovery in cognitive and emotive domains. All questionnaire responses in these domains yielded three dominant trajectories. Trajectories with single asterisk (*) denotes the best recovery trajectory and those with double asterisks (**) denote the worst trajectory. Colors are used for visualization purposes and do not correspond to better or worse trajectories. Patients’ level of anxiety (**a**), restless feeling (**b**), depressed feeling (**c**), rested feeling (**d**), being able to remember things (**e**), and being able to make decision quickly (**f**) are displayed.

**Fig. 3:**
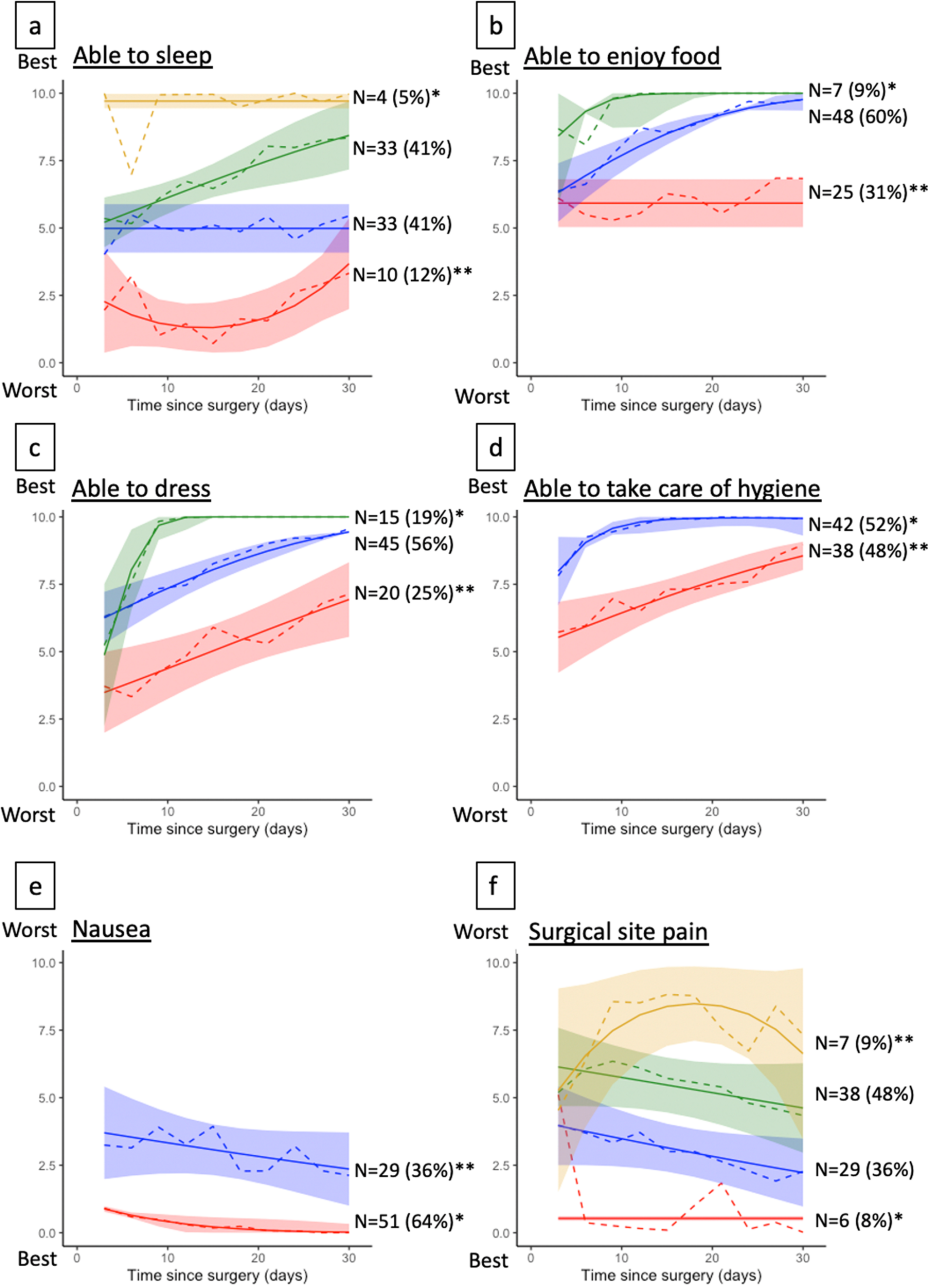
Trajectories of recovery by sleep, food, activities of daily living, and nociception. The figure shows trajectories of recovery in sleep, ability to enjoy food, activities of daily living, nausea, and pain. All questionnaire responses in these domains yielded three dominant trajectory groups, except for nausea and hygiene, which yielded 2 trajectory groups, and pain and sleep, which yielded four trajectory groups. Trajectories with single asterisk (*) denotes the best recovery trajectory and those with double asterisks (**) denote the worst trajectory. Colors are used for visualization purposes and do not correspond to better or worse trajectories. Patients’ ability to sleep (**a**), ability to enjoy food (**b**), ability to dress (**c**), ability to take care of hygiene (**d**), feeling of nausea (**e**), and surgical site pain (**f**) are displayed.

Group-based trajectory models for 12 questionnaire responses yielded trajectories shown in Figs. 2 and 3. The best-fit models classified patients into 3 groups for all questionnaires, except for surgical site pain and sleep, which yielded 4 trajectory groups, and hygiene and nausea, which yielded 2 trajectory groups. All the trajectory groups had a mean posterior probability of assignment of 0.80 or higher, indicating a good separation of trajectory class.

### Patient characteristics by the overall perception of recovery trajectories

Comparing patients who were classified into the worst overall recovery trajectory group (*n* = 11) versus the remainder who were classified into one of the three better trajectory groups (*n* = 69), there were no statistically significant differences across preoperative patient characteristics and surgical characteristics. Patients in the worst trajectory group had a higher incidence of postoperative complications: 24 patients (35%) experienced at least one complication in the better overall recovery groups, whereas 9 (75%) had complications among those in the worst overall recovery trajectory (*p* = 0.023 by chi-squared test). Postoperative length of stay was not significantly different. Among the complications, prolonged ventilatory support and reoperation for bleeding were statistically significantly higher in the worst overall recovery trajectory group (Table 1).

### Relationship between the overall recovery and other domains

From the trajectory groups identified in the 12 questionnaire responses in the other recovery domains, we graphically identified the worst trajectory in each of the 12 questionnaire responses (Figs. 2 and 3). Comparing the frequencies of belonging to the worst trajectories in each of the 12 items, only the worst anxiety trajectory was significantly more frequent in those belonging to the worst overall recovery trajectory (Table 2).

**Table 2:** Associations between belonging to the worst overall recovery trajectory vs. worst trajectories in other domains.

Worst trajectories in each domain	Good recovery (*N* = 68)	Worst recovery (*N* = 12)	*P*
Pain	7 (10%)	0 (0%)	0.6
Anxiety	6 (8.8%)	4 (33%)	**0.038**
Able to make decision	5 (7.4%)	2 (17%)	0.3
Depression	14 (21%)	4 (33%)	0.5
Able to dress	16 (24%)	4 (33%)	0.5
Able to enjoy food	19 (28%)	6 (50%)	0.2
Able to manage hygiene	30 (44%)	8 (67%)	0.15
Nausea	24 (35%)	5 (42%)	0.7
Able to remember	9 (13%)	2 (17%)	0.7
Feeling rested	31 (46%)	6 (50%)	0.8
Feeling restless	28 (41%)	5 (42%)	>0.9
Sleep	7 (10%)	3 (25%)	0.2

The table shows bivariate comparisons of the frequencies belonging to the worst trajectory in each of the displayed recovery domains, comparing the frequencies among those belonging to the worst overall recovery trajectory versus any of the better overall trajectory groups. The bolded value indicates *p* < 0.05. For significance testing, the chi-squared test and Wilcoxon rank-sum test were used for categorical and continuous variables, respectively.

## Discussion

In this descriptive study, we demonstrated that measurement of PROM as frequently as every 3 days yielded a good response rate using a digital platform, and the resulting data separated into distinct trajectory groups across various recovery domains based on latent class trajectory models. We also demonstrated that a patient’s sense of overall recovery may be associated with complications, but did not observe an association between the patient’s overall sense of recovery and any component domain of recovery, such as pain or ADLs. Although the evaluated associations may be limited by the sample size, this information may be used to set expectations and alleviate the worries of patients and families at preoperative counseling. Such recovery trajectory data may serve as indicators that could guide efforts to improve the post-acute recovery.

This study extends the literature in several ways. Although there are extensive data and models to predict mortality and complications in the first 30 days after cardiac surgery^11^, we have minimal understanding of the experience of patient recovery during this period^4^. While surgical and anesthesia societies recommend patients ask their doctors about the expected recovery course after surgery^12,13^, data to guide answering this question are lacking. For example, a systematic review on the postoperative recovery after cardiac surgery identified that studies evaluating short-term postoperative recovery at a high measurement frequency are rare and that the reporting methodology did not account for underlying heterogeneous trajectories^4^. Our study shows the feasibility of quantifying recovery via multi-domain measurement of PROM and reporting in a small number of trajectory groups to facilitate interpretation. Our analysis also demonstrated that even in a cohort of patients with low complication rates and no operative mortality, the recovery course, as experienced by the patients and measured by PROMs, varied substantially. This highlights the potential utility of PROMs to characterize the patient’s recovery course beyond conventional, observer-reported outcomes of mortality and postoperative complications. The findings suggest that digital collection and transmission of recovery data could play a vital role in the evaluation and management of recovery and in the pursuit of research to develop management strategies that improve outcomes.

To assess the feasibility of broader clinical implementation of frequent PROM data collection, our enrollment and follow-up protocols were designed to minimize the time and effort to enroll the patients and collect the PROM data. Leveraging a digital platform for survey delivery, reminder, and data collection and organization, we reduced the direct encounter between the investigator and participants to a single encounter for enrollment. In clinical practice, this enrollment may be performed by a clinician as part of the initial transfer assessment out of the intensive care unit (ICU). Future studies could explore the possibility of fully digital enrollment without direct patient-clinician encounters, as some trials have done. Expanding this approach to other surgical and interventional fields may offer further insights into a variable recovery based on the magnitude of the procedure.

Symptom monitoring alone has been shown to improve QoL in patients undergoing cancer treatment^14^. Our work provides a step towards broader implementation of PROM data collection and evaluating the impact of interventions on postoperative recovery. We used this method to identify a group of patients belonging to a particular postoperative pain trajectory that may benefit from earlier postoperative follow-up^15^. It is plausible that such information could be an early indicator of recovery and identify people who would respond to more intensive postoperative care.

We demonstrated that complications, specifically prolonged ventilatory support and return to the operating room for bleeding, were associated with a worse overall perception of postoperative recovery. While both complications are known to increase the length of stay^16^, how they may relate to the patient’s perception of recovery has been unknown. The Society of Thoracic Surgeons’ risk calculators provide predictions for the risk of both bleeding and prolonged mechanical ventilatory support based on variables that are available preoperatively. Therefore, such risks may be extrapolated to provide patients with the expectation for overall recovery from the patient’s perspective during preoperative counseling.

Our study identified a potential association between anxiety trajectory and overall recovery trajectory but did not find a clear association between the overall recovery trajectory and recovery in other domains, including pain, ADL, or sleep. Therefore, although patients may have persistent pain, limited ability to perform ADL, or poor sleep, the patient may still feel progression in their overall recovery. This also highlights the complexity of the perception of patient recovery and the importance of understanding and evaluating these using PROMs.

The single-center design of our study may limit the generalizability of our findings, although the variation in the phenotype of pain trajectories may be a finding applicable to practices in care settings different from ours. The sample size was limited due to the premature termination of the study related to the COVID-19 pandemic, restricting our ability for split-sample testing of trajectories and to make a more robust inference for characteristics associated with specific recovery trajectory, including multivariable analysis and evaluation of the recovery specific to the type of operations the patient underwent. The small sample size also likely increased the chance of type II errors. Post-hoc power analysis was not performed as the utility of such analysis to inform the chance of type II error is limited^17–19^. As expected, many patients did not complete all 10 delivered surveys. We delivered a high number of surveys to capture at least 3 responses for the trajectory to be modeled in the latent class analysis and demonstrated a reasonable response rate. We did not perform statistical corrections for multiple testing. Therefore, there may be an elevated chance that the statistically significant association observed is due to a Type I error, although there is a counterargument to routinely performing such corrections for multiple testing^20^. External validation of the trajectories could not be performed for the lack of a separate dataset.

Patient-reported information related to postoperative recovery after cardiac surgery can be measured using a digital platform with PROM questionnaires delivered at high frequency in a short postoperative interval. Perception of recovery varies even among a group of patients with low complication rates and no mortality.

## Methods

### Patient selection criteria and data source

**Fig. 4:**
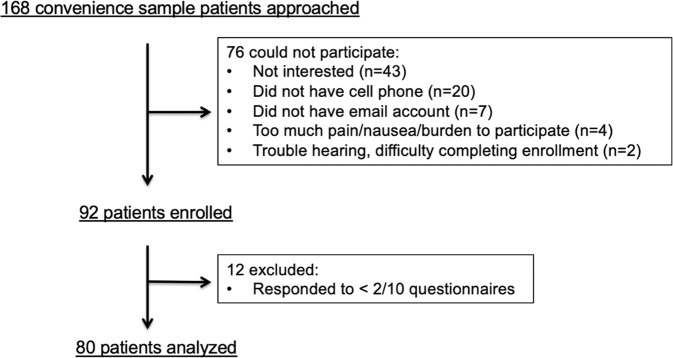
CONSORT-style enrollment flow chart. This figure outlines the enrollment flow. Of the 92 patients enrolled, 12 were excluded due to the low response rate precluding the trajectory analysis, resulting in 80 patients analyzed for this study. There was no mortality during the follow-up period.

We studied a convenience sample of patients who underwent cardiac surgery at Yale New Haven Hospital between January 2019 and March 2020. Yale New Haven Hospital is a tertiary center in the United States, where over 1100 cardiac surgeries are performed annually. Postoperative, as opposed to preoperative, enrollment allowed us to enroll patients undergoing non-elective surgery to reduce selection bias. Inclusion criteria were patients undergoing isolated or concomitant coronary artery bypass graft (CABG), aortic valve replacement, mitral valve replacement, mitral valve repair, or aortic operation who were discharged from the ICU within 5 days of the operation. This 5-day threshold ensured that the time of initiation of QoL assessments would be standardized since patients could not be enrolled in the ICU due to logistical challenges. A research assistant (RA) visited the patient and after confirming the patient was eligible to participate and following the description of the study procedure, obtained written informed consent from all study participants. Patients provided signed informed consent. We excluded patients who could not complete enrollment, did not own a smartphone or a tablet, and those who did not speak or read English because the electronic platform for PROM data collection relied on patients responding to surveys received via email or text and which opened on a web browser. We screened 168 patients, of which 92 (54.8%) met the eligibility criteria and were enrolled. We excluded 12 patients who responded only once, resulting in 80 who were analyzed for this study (Fig. 4).

Details of the protocol have been published^9^. The cardiac surgery service did not have a formalized Enhanced Recovery After Surgery (ERAS) pathway at the time of the study. Pain regimens were individualized to the patient’s needs during the hospitalization and at discharge. The Yale Institutional Review Board approved the study (IRB # 2000025689).

### Questionnaire and data collected

Quality of Recovery (QoR-24), a 24-item questionnaire assessing postoperative recovery^21–23^ adapted from the original QoR-40^24^, was delivered every 3 days for 30 days. The questionnaire for each item asked patients about the frequency of symptoms, ranging from 0 to 10, with 0 being ‘none of the time’ and 10 being ‘all of the time.’ For example, the questionnaire for pain read, ‘During the last 24 h, I have been having pain in the surgical wound:’ with possible responses ranging from 0 to 10 (Supplemental Figs. 1 and 2). Other domains of recovery assessed were: sleep, ADLs, nausea, feeling of depression, anxiety, feeling rested, feeling restless, ability to enjoy food, ability to make a decision quickly, being able to remember things, and patient perception of overall recovery (scored 0–100%, with 0% being not recovered at all and 100% being fully recovered).

Patient characteristics, intraoperative, and postoperative variables were prespecified^9^ and collected via the institutional Society of Thoracic Surgeons (STS) Adult Cardiac Surgery Database using the data version 2.91 definitions^25^.

### Digital platform characteristics

A patient-specific survey account was made during enrollment using the patient’s email address. We used HugoHealth, a digital health platform that integrates patients’ healthcare records, customization of survey deliveries, and organization of survey response data. The questionnaire was not integrated into the electronic health record system, which ensured the survey response did not elicit changes in treatment. Mobile devices used to respond to the questionnaires were patients’ own devices. The author (H.K.) is a co-founder of HugoHealth.

Electronic survey delivery via email, reminder, and response collection were all automated. Therefore, investigators directly contacted each patient only once during the study period, at the time of enrollment. To reflect the possible clinical implementation of survey delivery, we tested automated survey delivery and reminders instead of researchers contacting the patient to deliver the survey to ensure participation. After all the surveys were delivered, we asked patients with a low response rate (<5/10 surveys returned) and categorized the reasons they provided.

### Statistical analysis

We applied a group-based trajectory model, a family of latent class analyses, which estimated the probability of belonging to a specific QoL trajectory^26,27^. This is a semiparametric finite mixture model for longitudinal data using a maximum likelihood method fitting the survey response values with a censored normal distribution. We fitted the model from one to five trajectories with polynomial order of up to a cubic term. Attrition from the study was not modeled together, as there was no mortality during the study period.

We determined the optimal number of trajectory classes based on the BIC and average posterior probability of assignment (>0.9 indicated excellent fit and <0.7 indicated poor fit) among the models with one to five trajectory classes and incrementally increasing the polynomial order^28^.

To facilitate clinical interpretation of trajectories in each domain, trajectory groups identified via the latent class models were further dichotomized into one trajectory that distinctively indicates worse recovery than the rest. The worst trajectory class was identified graphically. We defined patients belonging to the worst overall recovery trajectory as the exposure of interest and compared patient characteristics and surgical and postoperative characteristics between those belonging to the worst overall recovery trajectory and all remaining patients belonging to better overall recovery trajectories.

For comparing patient characteristics between patients belonging to the worst versus the remaining overall recovery trajectory groups, we used Wilcoxon rank-sum test for continuous variables and the chi-square test for categorical variables. We also evaluated the association between belonging to the worst overall recovery trajectory groups and belonging to the worst recovery trajectories in other domains using chi-squared analysis. This analysis was performed to identify other recovery domains associated with the overall perception of recovery. We defined *p* = 0.05 as the threshold for statistical significance. We used the Traj package for a group-based trajectory model in SAS 9.4 (SAS Institute, Inc Cary, NC). The analysis was conducted by the first author (M.M).

### Missing data

We did not impute questionnaire responses. With ≥2 responses, the group-based trajectory model’s full information maximum likelihood estimation allowed for integrating all available information based on missing-at-random assumption^29^. Missing data for the STS data occurred in <2% of participants, and missing values were conditionally estimated as described by Shahian et al.^30^ in the STS risk model development, classifying missing values to those in the lowest risk category for categorical variables and using age and sex-specific means for continuous variables.

### Reporting summary

Further information on research design is available in the Nature Research Reporting Summary linked to this article.

## Supplementary information

Reporting Summary

Supplemental Figures and Tables

## Data Availability

The data that support the findings of this study are not openly available due to participant privacy. De-identified data are available from the first author upon reasonable request.

## Code availability

The code used for the analysis is available at 10.5281/zenodo.7316275.
