# Supplementary material for: Characterization of multi-domain postoperative recovery trajectories after cardiac surgery using a digital platform
Source: NPJ Digit Med. 2022 Dec 24;5:192. doi: 10.1038/s41746-022-00736-0 (PMC9789027; doi:10.1038/s41746-022-00736-0)
Supplement: Supplementary file 2 — Supplemental Figures and Tables [file 41746_2022_736_MOESM2_ESM.pdf]

Supplemental Figure and Table

Supplemental Table 1: Patient characteristics by the survey response rate

Characteristics	Response <80% (N = 36)	Response >= 80% (N=44)	P
Worst recovery class	3 (8.3%)	9 (20%)	
Age	62 (57, 70)	65 (58, 71)	0.4
Woman	9 (25%)	11 (25%)	>0.9
Race			0.9
Black	1 (2.8%)	3 (6.8%)	
White	33 (92%)	39 (89%)	
Other	2 (5.6%)	2 (4.5%)	
Diabetes	14 (39%)	12 (27%)	0.3
Hypertension	25 (69%)	31 (70%)	>0.9
Liver disease	3 (8.3%)	3 (6.8%)	>0.9
Creatinine (mg/dL)	0.97 (0.83, 1.20)	0.93 (0.84, 1.10)	0.6
Cerebrovascular disease	5 (14%)	4 (9.1%)	0.7
Prior myocardial infarction	10 (28%)	13 (30%)	0.9
Heart failure	14 (39%)	11 (25%)	0.2
Ejection fraction (%)	60 (54, 63)	59 (55, 63)	0.8
Status			>0.9
Elective	25 (69%)	31 (70%)	
Urgent	11 (31%)	13 (30%)	
Re-do operation	4 (11%)	3 (6.8%)	0.7
CABG	17 (47%)	23 (52%)	0.7
Aortic surgery	1 (2.8%)	3 (6.8%)	0.6
AVR	11 (31%)	7 (16%)	0.12
Mitral procedure	25 (69%)	28 (64%)	0.9
Any complications	15 (42%)	18 (41%)	>0.9
Surgical site infection	3 (8.3%)	0 (0%)	0.087
Reoperation for bleeding	1 (2.8%)	1 (2.3%)	>0.9
Sepsis	0 (0%)	0 (0%)	
Stroke	0 (0%)	0 (0%)	
TIA	0 (0%)	0 (0%)	
Prolonged ventilatory support	1 (2.8%)	1 (2.3%)	>0.9
Pneumonia	0 (0%)	1 (2.3%)	>0.9
VTE	1 (2.8%)	0 (0%)	0.4
Renal failure	0 (0%)	0 (0%)	
Readmission	4 (11%)	2 (4.5%)	0.4

The table shows comparisons of patient characteristics by those who responded 80% or more of the surveys versus those responded less than 80%. For significance testing, chi-squared test and Wilcoxon rank-sum test were used for categorical and continuous variables, respectively. CABG = coronary artery bypass graft surgery; AVR= surgical aortic valve replacement; MV = mitral valve; TIA= transient ischemic attack; VTE = venous thromboembolism.

Supplemental Figure 1: Screenshot of a delivered questionnaire

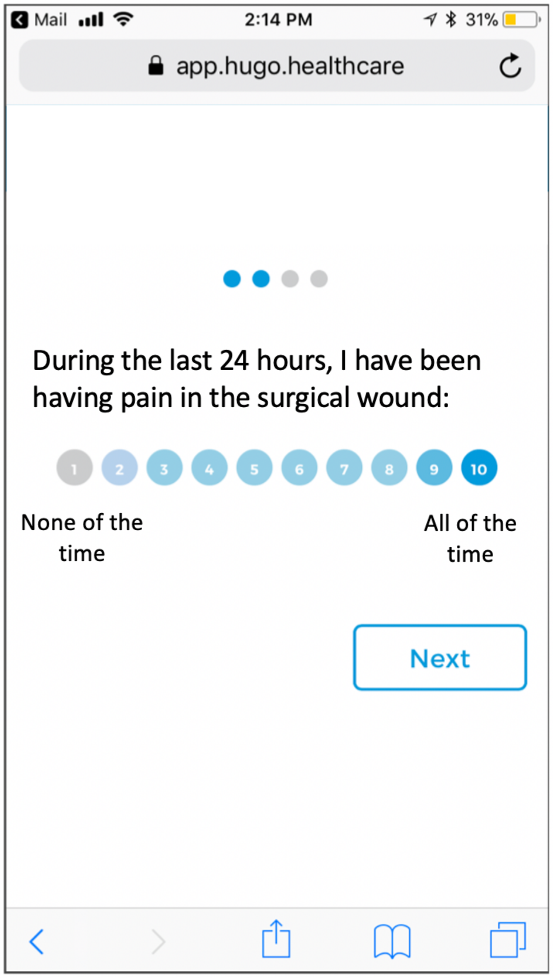

The figure shows a screenshot example of a questionnaire delivered to the participants.

Supplemental Figure 2: Modified Quality of Recovery (QoR-24) Questionnaire

Modified Quality of Recovery (QoR-24) Questionnaire

*Answered in visual analogue scale: 0 [none of the time] to 10 [all of the time]
'During the last 24 hours, I have been…'

1. Able to breathe easily
2. Having normal bowel function
3. Able to enjoy food
4. Speaking normally
5. Able to think clearly
6. Able to remember things
7. Able to make decisions quickly
8. Able to take care of own hygiene
9. Able to write
10. Able to dress easily
11. Having pain in the surgical wound
12. Having nausea
13. Shivering or twitching
14. Feeling dizziness
15. Feeling restless
16. Feeling rested
17. Feeling depressed
18. Feeling lonely
19. Having anxiety
20. Sleeping well
21. Difficulties getting to sleep
22. What time did you fall asleep? What time did you wake up without going back to sleep?
23. How much do you think you have recovered? (0-100%)
24. Open ended question: 'Please describe anything bothersome to you in terms of emotion, thinking, sleep, mobility, discomfort, carrying out daily activity, or symptoms.'
